# Calcium Input Frequency, Duration and Amplitude Differentially Modulate the Relative Activation of Calcineurin and CaMKII

**DOI:** 10.1371/journal.pone.0043810

**Published:** 2012-09-04

**Authors:** Lu Li, Melanie I. Stefan, Nicolas Le Novère

**Affiliations:** EMBL European Bioinformatics Institute, Hinxton, United Kingdom; University of Toronto, Canada

## Abstract

NMDA receptor dependent long-term potentiation (LTP) and long-term depression (LTD) are two prominent forms of synaptic plasticity, both of which are triggered by post-synaptic calcium elevation. To understand how calcium selectively stimulates two opposing processes, we developed a detailed computational model and performed simulations with different calcium input frequencies, amplitudes, and durations. We show that with a total amount of calcium ions kept constant, high frequencies of calcium pulses stimulate calmodulin more efficiently. Calcium input activates both calcineurin and Ca^2+^/calmodulin-dependent protein kinase II (CaMKII) at all frequencies, but increased frequencies shift the relative activation from calcineurin to CaMKII. Irrespective of amplitude and duration of the inputs, the total amount of calcium ions injected adjusts the sensitivity of the system to calcium input frequencies. At a given frequency, the quantity of CaMKII activated is proportional to the total amount of calcium. Thus, an input of a small amount of calcium at high frequencies can induce the same activation of CaMKII as a larger amount, at lower frequencies. Finally, the extent of activation of CaMKII signals with high calcium frequency is further controlled by other factors, including the availability of calmodulin, and by the potency of phosphatase inhibitors.

## Introduction

NMDA receptor dependent long-term potentiation (LTP) and long-term depression (LTD) are two forms of activity-dependent synaptic plasticity, a process at the origin of learning and memory [1], [2]. It has been shown that high frequency of synaptic stimulation leads to LTP [3], while low frequency stimulation results in LTD [4]. In both cases, stimulation triggers postsynaptic membrane depolarization, which leads to the activation of synaptic NMDA receptors, and the subsequent elevation of intracellular calcium concentration. Calcium, via calmodulin, activates Ca^2+^/calmodulin-dependent protein kinase II (CaMKII), inducing LTP [5], [6], or calcineurin, triggering LTD [7].

It has been proposed that substantial increases in postsynaptic calcium concentration selectively activate CaMKII, while moderate rises activate calcineurin [8]–[11]. However, several observations suggest that this hypothesis is inadequate. First of all, intracellular calcium level increases in the form of spikes rather than by gradually reaching a steady level. This is due to both the large number of calcium binding proteins, which act as calcium buffers, and to calcium efflux mechanisms, which function to lower calcium concentration to basal level within a few hundred milliseconds [12]. These fast calcium transients suggest that the increase in calcium level depends not only on the amplitude of each input, but also on the frequency and duration of inputs. Second, a brief sub-molar increase of calcium has been found to trigger LTP and LTD with similar probabilities [13]. Third, different temporal patterns of postsynaptic calcium elevation have been shown to selectively induce LTP or LTD [14]. Taken together, this evidence suggests that the temporal patterns of calcium increase, rather than its amplitude, are the key signal carrying significant biological information. The question remains, however, as to how signaling pathways are able to decipher the temporally-encoded calcium signals through key signaling molecules such as calmodulin, or CaMKII.

Calmodulin, an important calcium-dependent regulatory protein, possesses four EF-hand calcium binding domains [15], and can exist in two distinct conformations: the closed (or tense, T) state [16] and the open (or relaxed, R) state [17]. Calcium cooperatively binds to calmodulin [18]. The binding of four calcium ions is not necessary for calmodulin function, since unsaturated calmodulin can also activate its targets [19]. Stefan *et al.* proposed an allosteric model for calmodulin activation, illustrating how the binding of calcium ions progressively stabilizes the high affinity R state [20]. Furthermore, in this model, calmodulin can differentially activate calcineurin and CaMKII according to static calcium concentration values. This raises the question of whether this allosteric device is also able to decode patterns of calcium spikes.

Calcineurin is the only known protein serine/threonine phosphatase that is directly regulated by Ca^2+^ and Ca^2+^/calmodulin [21], [22]. Calcineurin is a heterodimer, consisting the catalytic A subunit and the regulatory B subunit. Its phosphatase activity depends not only on the binding of Ca^2+^/calmodulin to calcineurin A, but also the binding of calcium ions to calcineurin B, which contains four EF-hands structures [23], [24]. Calcineurin exerts its effect on synaptic plasticity not only through its direct role of dephosphorylating residue Ser845 on the GluR1 subunit of AMPA receptors [25], but also through dephosphorylating, and thus inactivating, protein phosphatase 1 (PP1) inhibitors (I1 or DARPP-32) [26]. PP1 can also dephosphorylate AMPA receptor [27]. Besides, PP1 dephosphorylates CaMKII on threonine 286 (Thr286), providing a mechanism of negative regulation of CaMKII by calcineurin.

The CaMKII holoenzyme is a dodecamer structure composed of two rings of hexamers [28], [29]. Binding of calmodulin to monomeric CaMKII subunit stabilizes its activity [30]. CaMKII has been shown to cooperatively bind calmodulin [31], [32]. CaMKII can autophosphorylate on Thr286. This autophosphorylation requires the binding of calmodulin and the catalytic ability from an active neighboring subunit within the same hexameric ring [33], [34]. Phosphorylated CaMKII then remains in a constitutively active state, independent of calmodulin binding [33], [35], unless being dephosphorylated by PP1. The autophosphorylation increases the apparent affinity of calmodulin binding to CaMKII [36]. CaMKII autophosphorylation has been shown to be a decoder of calcium spike frequency *in vitro* [37]. However, many questions remain open: How does CaMKII respond to calcium inputs in the presence of phosphatases? What is the relative activity of CaMKII and calcineurin during high frequency calcium oscillations?

To answer these questions, detailed quantitative computational models are required since biochemical experiments are often constrained, for example, by the limitations of available chelators able to reveal different patterns of calcium spikes [14]. Some progress has been made; the calcium sensitivity of CaMKII has been studied in many mathematical models [38]–[42]. The calcium spike pattern dependent activation of post-synaptic signaling pathways has been discussed [43]–[47]. Finally, the dependence of calmodulin activation on calcium spike frequency has been investigated [48], [49].

However, these models show limitations. First of all, the activation of calmodulin has been either ignored [38] or simplified. A common strategy for simplification is to model instantaneous calmodulin activation as a function of calcium concentration, either in the form of Hill or Adair-Klotz equations [40], [41], [43], [45], thus ignoring the dynamics of calcium binding and release from calmodulin. Another approach is to model calmodulin activation by sequential binding of four calcium ions, often regardless of the different affinities and cooperativity among the four binding sites [39], [46]–[51]. Secondly, in these models phosphatase activity, and most importantly the regulation of these phosphatases by Ca^2+^/calmodulin, have either been ignored [38]–[41] or not modeled in detail (for instance the ability of calcium ions to bind to the calcineurin B subunit) [43], [50], [51]. Thirdly, when postsynaptic calcium increase was considered as model input, calcium has been, in general, modeled as an exponential or a sinusoidal function. Therefore, calcium concentration decays passively, which does not reflect the changes caused by calcium binding process [38], [40]–[43], [51]. In addition, the increase and decay of calcium pulse demonstrated by these models do not correspond to the changes of intracellular free calcium spikes, and can not compare with experimental observations. Some models do not distinguish the frequency of calcium spikes from the total amount of calcium ions. Since these models applied the same stimulation duration for both low-frequency and high-frequency calcium spikes, the low-frequency calcium input actually means smaller quantity of calcium ions, comparing with high-frequency calcium input [39]. Finally and most importantly, as far as the authors know, there is no model that systematically compares the activities of phosphatase (calcineurin) and kinase (CaMKII) upon stimulation of different calcium spike frequencies, while keeping the total amount of calcium ions constant.

**Figure 1:**
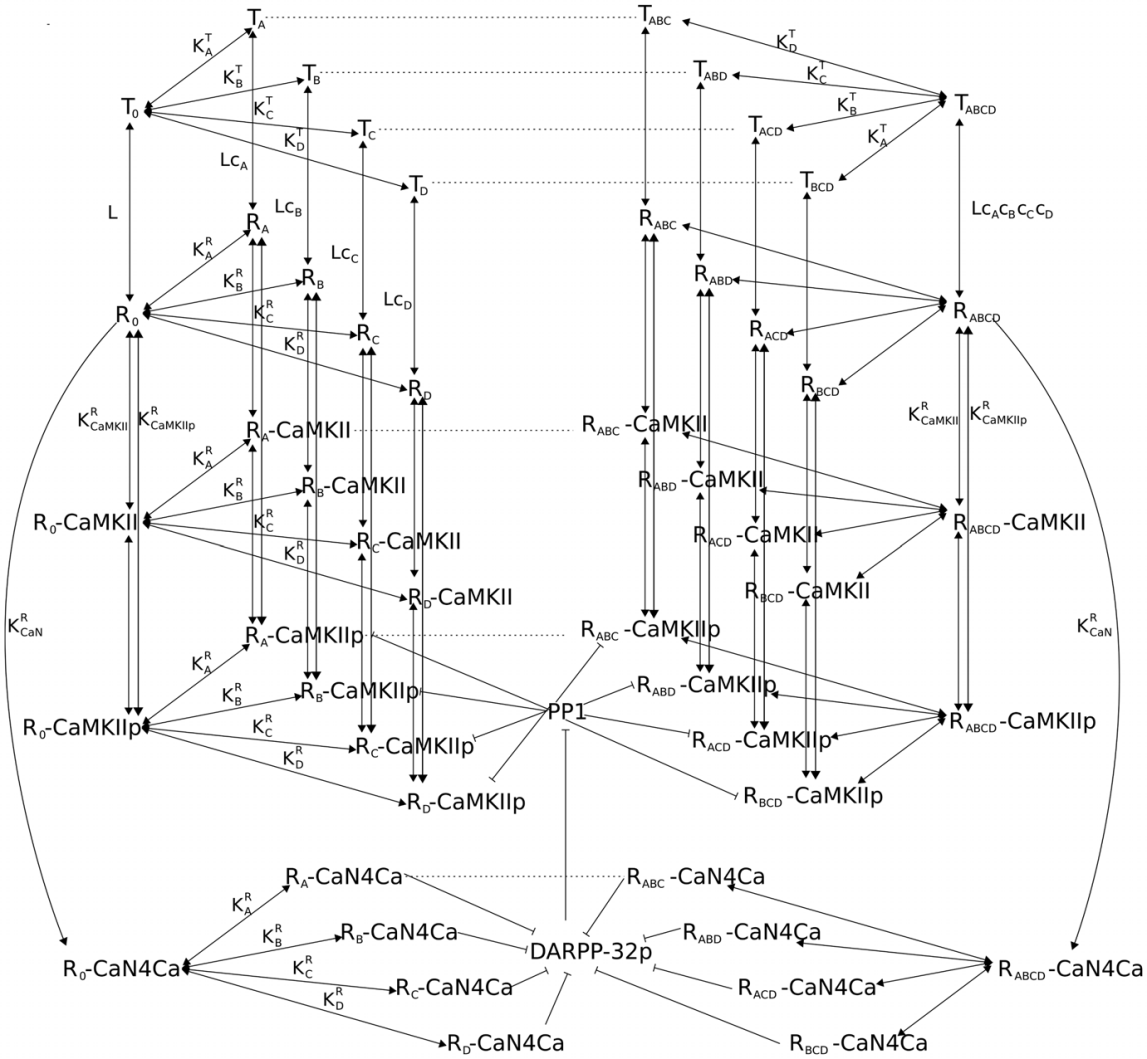
Reaction diagram of calmodulin regulated pathways. Graphical representation of the model implemented in this study. Part of this model is based on a published allosteric model of calmodulin function [20]. For clarity, only the first and fourth calcium-binding-calmodulin events and their related reactions are depicted in detail (dashed lines indicate second and third calcium-binding events). Filled arrow: yield, bar arrow: inhibition or dephosphorylation, R: calmodulin in active state, T: calmodulin in inactive state, subscripts following T or R: calcium binding sites, CaN: calcineurin, DARPP-32p: phospho-DARPP-32 at Thr34, K: dissociation constant, L:allosteric equilibrium constant, c: the ratio of dissociation constants for R and T states.

The study presented here is based on a published allosteric model of calmodulin [20]. In this model, Stefan *et al.* depicted various properties of calmodulin, including the cooperativity of calcium binding, different affinities for calcium binding sites, and the activity of calcium-unsaturated calmodulin. The authors also proposed that the differential activation of calcineurin and CaMKII is based on the static concentration of calcium elevation. However, this model does not take into account the binding of calcium ions to the regulatory subunit of calcineurin, the autophosphorylation of CaMKII, and the negative regulation by calcineurin of the activation of CaMKII. Most importantly, the activation of calcineurin and CaMKII by calcium spikes has not been assessed. We expanded the model of Stefan et al. to include inter-holoenzyme autophosphorylation of CaMKII, using a rate based on the probability of having an active neighboring subunit at each simulation step. The activation of calcineurin by binding calcium ions and activated calmodulin has also been modeled in greater detail. In addition, we included reactions describing the dephosphorylation of CaMKII by PP1, the inhibition of PP1 by DARPP-32, and the dephosphorylation of DARPP-32 by calcineurin (Figure 1). We modeled the calcium spikes according to experimental measurements [12], with explicit binding and dissociation reactions involving calcium buffer proteins. We systematically compare the effects of calcium input frequency, duration and amplitude on the activities of both CaMKII and calcineurin.

## Results

### Modeling calcium spikes and simulation design

**Figure 2:**
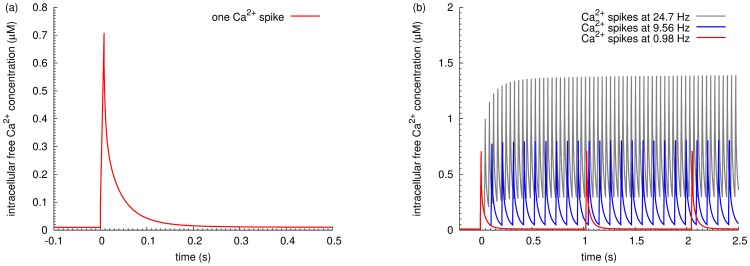
Intracellular free calcium concentration increase induced by stimulation inputs. Increase of postsynaptic free calcium concentration triggered either by a single calcium input or by a train of inputs. (a) With a single input (34560 molecules), intracellular free calcium reaches maximal level, 0.7 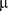
M (corresponding to 421 molecules), within 10 milliseconds, and decays back to basal level within 200 milliseconds. (b) Intracellular free calcium concentration increases after a train of inputs at 0.98 Hz (red line), 9.56 Hz (blue line), and 24.7 Hz (grey line). As the frequency of calcium inputs increases, the free calcium concentration rises both at its basal level and its maximal value. Note: The plotted duration does not correspond to the simulation time.

The transient changes of free calcium concentration in the spine are shaped by many factors including calcium sources, calcium extrusion mechanisms, and distribution of calcium buffer proteins. In this study, we focused on the calcium spikes induced by synaptic stimulation. Using the model described in the methods section, we showed that a single calcium input of 34560 molecules induced free intracellular calcium transients reaching the peak level of 0.7 micromolar (corresponding to 421 molecules), within 10 milliseconds, followed by a decay to basal levels within 220 milliseconds (Figure 2a). Such a spike is in agreement with the amplitude and time course of NMDA receptor mediated calcium transients in an individual spine in partially depolarized conditions [12]. This single input was repeated to induce a train of calcium spikes, with varied intervals, to form signals with different frequencies (in the following text, a single “input” induces one free intracellular calcium spike, while the “inputs” triggers a series of free intracellular calcium spikes at corresponding frequency).

First, we modulated the calcium signal purely on frequency, without changing the number of inputs or the input size. This generated either a prolonged low frequency stimulation, or a relatively short-lived high frequency stimulus. In total, 41 different frequencies, ranging from 0.1 Hz to 200 Hz, were studied. For each frequency, 100 calcium inputs were created after the system reached steady state (800 seconds after initiation of the simulation. Therefore, while the stimulations contained calcium inputs at different frequencies, equal amounts of calcium ions were used.

A train of calcium inputs at around 1 Hz did not change the peak free calcium level (Figure 2b), when compared with a single input (Figure 2a). In contrast, a succession of inputs at around 10 Hz and 25 Hz gradually raised not only the peak free intracellular calcium level but also the basal value (Figure 2b). At 50 Hz, the free calcium concentration reached around 6.5 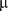
M (see Figure S1), which corresponds to the postsynaptic calcium increase observed in CA1 spines after stimulation of the Schaffer collateral [52].

Finally, the total number of calcium ions entering the spine was modulated in two ways. Firstly, the number of inputs varied from 10 to 180, the input size remaining the same. We compared 41 different stimulation frequencies for each input number. Secondly, the input size was changed, while the number of inputs was kept identical. For each input size, the same 41 different frequencies were tested.

### Effect of calcium input frequency on calmodulin activation

**Figure 3:**
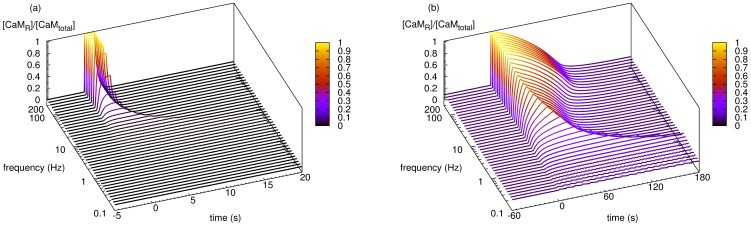
Effects of calcium input frequencies on the activation of calmodulin. Dependence of calmodulin activation on calcium input frequencies in models without or with targets. Each curve represents a time course of normalized active calmodulin, stimulated by a train of calcium inputs at a specific frequency. Although the frequency differs between curves, the total number of calcium inputs and the input size remain the same (100 inputs, 34560 molecules for each input). (a) Calmodulin activation in the model where calcineurin and CaMKII are not present. (b) Calmodulin activation in the model where calcineurin and CaMKII are present. From 10 to 70 Hz, the duration of calmodulin activation increases while, as the frequency increases, the duration of calcium stimulation decreases.

The frequency of the calcium signal played a crucial role in the activation of calmodulin. Figure 3b shows the time courses of calmodulin activation as a function of calcium inputs of varying frequencies, but equal amplitude. In basal conditions, less than 10% of total calmodulin was activated, and calcium inputs at low frequencies induced no significant change in the activation of calmodulin. This reflects the fact that calcium levels decayed before calcium bound significantly to calmodulin. However, as the frequency of calcium input increased, more calmodulin became active although for shorter periods, because the total duration of signal decreased. Starting from 10 Hz, the duration of calmodulin activation was prolonged as the frequency increased, although the signal duration was actually reduced. This shows calmodulin was trapped in the active state by its target, even after the departure of the calcium stimulation. This result could not be observed in a calmodulin model where calcineurin and CaMKII were not present (Figure 3a). At 50 Hz, almost 100% of the total calmodulin was in the active state at maximal activation, and more than half remained active for longer than 1 min (Figure 3b). As a result, frequencies that were higher than 50 Hz had little additional effect. At frequencies below 50 Hz, calmodulin was able to decode the calcium input frequency, and translated this information into the amount of active calmodulin, which was then transmitted to its targets. Moreover, those targets also stabilized its active state.

### Effect of calcium input frequency on calcineurin and CaMKII activation

**Figure 4:**
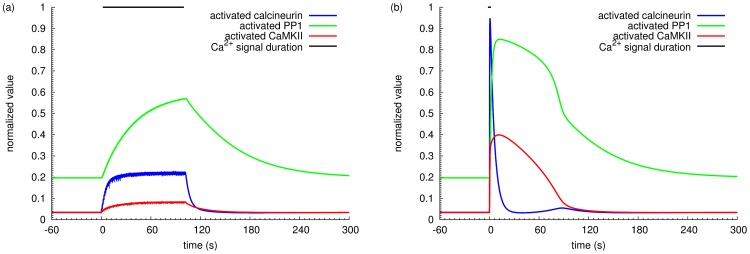
Effects of a train of calcium inputs on activation of calcineurin and CaMKII. Time courses of normalized activated CaMKII, calcineurin, and PP1 in response to a 100 input calcium stimulation at (a) 0.98 Hz, and (b) 52.8 Hz.

**Figure 5:**
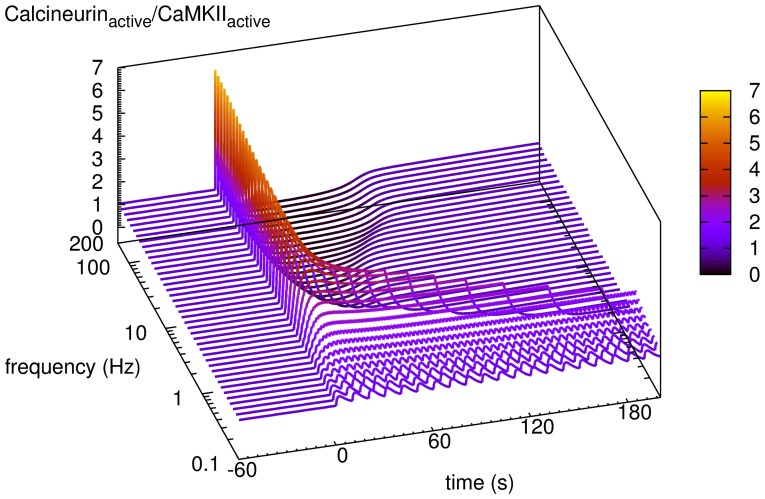
Comparison of calcineurin and CaMKII activation induced by calcium inputs at different frequencies. Dependence of the respective activation of calcineurin versus CaMKII on the frequency of calcium inputs. Each curve represents a time course of the ratio of activated calcineurin versus CaMKII, for a train of calcium inputs at a specific frequency. For each frequency, the stimulus is composed of the same number of inputs with the same input size (100 inputs, 34560 molecules for each input).

The relative activation of calcineurin and CaMKII dictates the direction of synaptic plasticity, since their respective activation can trigger opposing consequences on synaptic weight. Figures 4a and b show the time courses of calcineurin, CaMKII and PP1 in response to calcium inputs at two specific frequencies: 1 Hz and 50 Hz. In basal conditions, approximately 3% of total calcineurin was active, the same as CaMKII subunits, due to the low basal activity of calmodulin. Calcium inputs at 1 Hz induced more than 20% activation of calcineurin but less than 10% CaMKII at the peak level. Calcium inputs at 50 Hz triggered a high but transient activation of calcineurin, immediately followed by a modest but prolonged activation of CaMKII, despite the fact that activated calcineurin indirectly activated PP1 that dephosphorylated CaMKII on Thr286. When the activity of CaMKII reached peak level, calcineurin plunged down to slightly below basal level, indicating that CaMKII competes with calcineurin for active calmodulin. Figure 5 illustrates the time courses of the ratio of active calcineurin versus CaMKII, following different frequencies of calcium stimulation. Interestingly, from 0.1 Hz to 1 Hz, we can first observe an increased activity of calcineurin both in terms of amplitude and of duration. When frequency further increases, calcineurin activity further raises in amplitude, but with gradually shortened duration. At these frequencies, these calcineurin-to-CaMKII-ratio curves drop further down below the basal level, indicating the increased CaMKII activity immediately after the transient activation of calcineurin. It seems that high frequency calcium inputs do not prevent the activation of calcineurin. Instead, they change the dynamics of activities. The information carried by the frequency of the calcium signal is thus translated into the relative amplitude and duration of activation of calcineurin and CaMKII.

**Figure 6:**
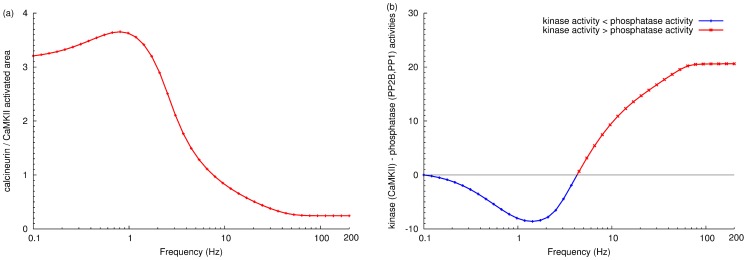
Calcium-spike-frequency dependent synaptic plasticity. (a) Ratio of calcineurin to CaMKII activated area. Each point represents the ratio of activated areas, upon the stimulation by a train of calcium inputs at a specific frequency (for the detailed calculation of “activated area”, see the methods section) (b) Difference between kinase (CaMKII) activity and phosphatase (calcineurin and PP1) activities. Enzyme activity is calculated as the product of an enzyme's activated area and its catalytic constant towards the GluR1 subunit of the AMPA receptor. Each point corresponds to a specific calcium spike frequency. For both graphs, stimulation at each frequency is composed of 100 calcium inputs with the same input size.

This complex interplay of duration and intensity of activation makes intuitive comparison of calcineurin and CaMKII activity unfeasible by sole examination of the timecourses. We therefore decided to assess the activation of calcineurin and CaMKII by looking at the integral of activation over time, a measure we call the “activated area” (for a detailed definition, refer to the methods section). Assuming the catalytic constant remain unchanged throughout the measurement, this area represents the “quantity of enzyme activity”. Figure 6a represents the ratio of the activated areas of calcineurin and CaMKII in response to calcium stimuli at 41 different frequencies, and suggests a frequency-dependent bidirectional response. A modest increase (0.1–1 Hz) of calcium-input frequency triggers an increase in calcineurin activity. However, a larger increase in frequency favored higher relative increases in CaMKII activity. In the 1–10 Hz spike frequency range, there was a sharp drop in the ratio of stimulated calcineurin to CaMKII, indicating increased CaMKII activation. This shift of activity became less dramatic when the frequency further increased, and stabilised at 50 Hz, where the activated area of CaMKII was more than four times that of calcineurin. Therefore, it is the frequency of the calcium signal that determines the relative activation of calcineurin and CaMKII, with low calcium stimulus frequencies preferring calcineurin, while high stimulus frequencies favor CaMKII activation.

To understand the biological impact of the frequency-regulated activity shift on synaptic efficacy, we need to understand how AMPA receptor is regulated by CaMKII, calcineurin and PP1. If we assume a fast equilibrium for the binding of AMPA receptor to these enzymes, its phosphorylation can be reflected by the activity of the kinases, while its dephosphorylation can be indicated by the activity of the phosphatases. Therefore, the efficacy of AMPA receptor could reflect the (mathematical) integration of the activities of the kinases and phosphatases [53]. Specifically, the evolution of phosphorylation on Ser831 and Ser845 of GluR1 subunit of the AMPA receptor follow a summation of the products of enzyme concentrations along time (the activated area) by their catalytic constants, phosphatase being considered as the “negative kinases”. As shown in Figure 6b, when we subtract the activities of calcineurin and protein phosphatase 1 from CaMKII, we could clearly observe a bi-directional regulation of synaptic plasticity, with three phases of regulation: 1) Increasing activity of phosphatases during low-frequency-calcium stimulation (below 2 Hz), indicating dephosphorylation on AMPA receptor; 2) Decreasing activity of phosphatases and/or rising activity of kinase (between 2 and 100 Hz approximately), reflecting the enhanced phosphorylation on AMPA receptor; 3) Saturation effect on kinase and phosphatases (beyond 100 Hz), due to the basal activity of phosphatase and/or lack of calmodulin (as discussed below).

### Effect of the total amount of calcium ions

**Figure 7:**
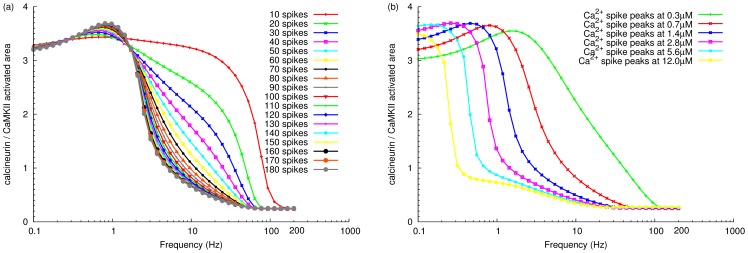
Effect of total amount of calcium ions on the relative activation of calcineurin and CaMKII. Comparison of the relative activation between calcineurin and CaMKII, stimulated by different numbers of calcium inputs, with the same input size (34560 molecules) (a), or different sizes of calcium input, with the same number of inputs (100 inputs) (b). For both graphs, each point represents the ratio of calcineurin versus CaMKII activated area, when stimulated by a train of calcium inputs at a given frequency.

Knowing that calcium signal frequency is crucial for the activation of calcineurin and CaMKII, we investigated the role of the absolute amount of calcium ions entering through NMDA receptors. We initially varied the total amount of calcium ions by changing the number of calcium inputs used in a stimulus, while maintaining the input size as previously described (Figure 2a). As can be seen in Figure 7a, at any given frequency above 2 Hz, CaMKII activation increased with the number of inputs. Moreover, increased input numbers shifted CaMKII activity towards lower frequency stimuli, while reduced input numbers required much higher frequency to activate CaMKII. However, when the number of inputs reached a certain threshold, the increased quantity of calcium ions could not further increase CaMKII sensitivity, even for low-frequency stimuli. This rules out a limiting role of calmodulin amount, because calmodulin activation hardly saturates at low input frequencies (Figure 3b). On the contrary, at any given frequency less than 2 Hz, increased spike number augments the peak calcineurin activity level instead of CaMKII, although in a less dramatic way when compared with the effect on CaMKII at higher frequencies. Irrespective of the number of calcium inputs, the ratio of active calcineurin versus CaMKII reached the same level at the 200 Hz signal, indicating that the final extent of CaMKII activation at high calcium input frequencies is independent of the number of inputs, i. e. the total quantity of calcium ions.

As discussed by Sabatini *et al.*, the amplitude of NMDA receptor dependent free calcium spikes depends on the depolarization of the postsynaptic membrane, the peak free calcium concentration ranging from 0.7 to 12 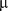
M [12]. A second approach to varying the total number of calcium ions in the stimulus was therefore to increase the size of each input while keeping the same number of inputs (100 inputs). Therefore, we varied the calcium inputs, to have different peaks of a free calcium spike ranging from 0.3 to 12 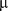
M. Each free calcium spike reached peak level within 10 milliseconds, and declined over approximately 200 milliseconds (see Figure S2), which is in agreement with experimental measurements [12]. As the amplitude of each calcium spike increased, the curve of relative activity between calcineurin and CaMKII shifted towards lower frequencies, indicating large calcium input size increased the proportion of active CaMKII at low frequencies (Figure 7b). At 200 Hz of calcium spikes, the final ratio of calcineurin versus CaMKII activity remained the same across different calcium input sizes, meaning that the level of activated CaMKII at high frequency stimulation is independent of the calcium input size. This situation is similar as when we varied calcium spike number. However, simulations with larger calcium input size reached this final ratio more rapidly than those with smaller input size. Sabatini *et al.* argued that NMDA-receptor-induced Ca^2+^ influx at resting membrane potential triggers LTD, but induces LTP when coupled with postsynaptic membrane depolarization because of the large amplitude of calcium input. Combined with our simulation results, a more complete picture might be that a large amplitude of calcium influx more easily induces LTP, because it relies less on high spiking frequencies.

Thus, high frequencies of calcium inputs provide a mechanism to produce transient but potent calcium elevations in order to activate CaMKII. This is important when each individual calcium influx is not large enough, or when the number of calcium inputs is limited. In parallel, a large amount of calcium ions entering into the spine can lower the threshold frequency required for strongly activating CaMKII. Therefore, the total amount of input calcium ions contributes to the sensitivity of the system towards decoding frequencies, and determines above which frequency CaMKII can overcome calcineurin, therefore when LTP can be triggered rather than LTD. However, the maximal extent to which CaMKII can be activated seems independent of the actual quantity of calcium ions, though it may depend on other factors, such as the availability of calmodulin, and phosphatase inhibitors.

### CaMKII autophosphorylation amplifies sensitivity of CaMKII towards calcium input frequency

The autophosphorylation of a CaMKII subunit on Thr286 requires that both this subunit and an adjacent neighbor are simultaneously active [33]. Upon this autophosphorylation, CaMKII acquires calcium-independent activity. In addition, the affinity of CaMKII for calmodulin markedly increases, a phenomenon known as calmodulin trapping [30], [36]. Thus, autophosphorylation locks CaMKII in the active state long after the end of a calcium elevation and favors CaMKII in the competition with other calmodulin-binding proteins [34], [54].

**Figure 8:**
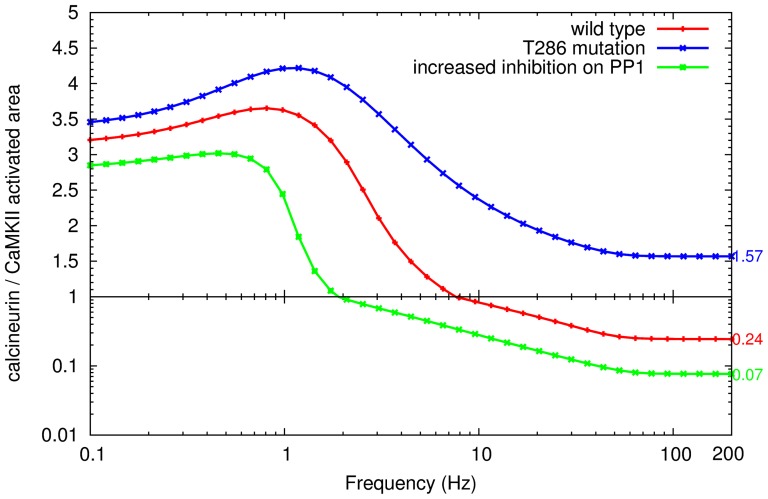
CaMKII autophosphorylation and increased PP1 inhibition on the relative activation of calcineurin and CaMKII. Ratio of calcineurin to CaMKII activated area for the wild type (red line), a CaMKII autophosphorylation mutant (at residue Thr286) (blue line), and the increased inhibition from DARPP-32 to PP1 (green line). The CaMKII autophosphorylation mutation was achieved by assigning the catalytic constant of phosphorylation as 0. For increasing the inhibition of DARPP-32 on PP1, the constant of competitive inhibition (ki) was reduced from 100 nM (in wild type) to 10 nM. Each point represents the ratio of activated areas, upon the stimulation by a train of calcium inputs at a specific frequency. Stimulation at each frequency is composed of 100 calcium inputs with the same input size (34560 molecules).

To understand the effect of this phosphorylation event, we studied the behavior of a phosphorylation-deficient *in silico* mutation by setting the autophosphorylation rate to zero. Interestingly, as shown in Figure 8, the activation of CaMKII still exhibits frequency sensitivity, since higher frequency decreases the ratio of calcineurin and CaMKII activities, indicating increased levels and duration of CaMKII activity. However, the activation of CaMKII at every frequency became less pronounced than in the wild type, and most importantly, mutated CaMKII attracted less calmodulin and hardly overcame the activation of calcineurin at higher frequencies. At lower frequencies, calcineurin activity increased more than in the situation when CaMKII autophosphorylation is intact. This means that autophosphorylation effectively influences the response of CaMKII to calcium inputs, and more specifically, that autophosphorylation on Thr286 strengthens the ability of CaMKII to compete for calmodulin with calcineurin. In addition, when the frequency of calcium inputs became higher, the divergence of responses between the mutated and wild-type CaMKII became wider, which shows that the autophosphorylation of CaMKII influences mostly its response to calcium inputs at higher frequencies. This can also be interpreted as CaMKII autophosphorylation being mainly triggered at higher frequencies of calcium inputs. To summarize, autophosphorylation on Thr286 deepens CaMKII sensitivity to calcium input frequencies, resulting in the display of more distinct activity levels. This autophosphorylation plays a crucial role in regulating the direction of synaptic plasticity.

DARPP-32 interacts with PP1 through two domains, which contribute to both low affinity binding and inhibition [55]–[58]. Upon binding both sites, the affinity between DARPP-32 and PP1 is increased to a nanomolar range [56], [59]. In our model, a relatively low affinity (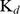 = 100 nM) was used, representing the average inhibitory effect of different PP1 inhibitors, under basal conditions. However, when the affinity between DARPP-32 and PP1 was increased by 10-fold (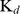 = 10 nM) [59], with basal DARPP-32 phosphorylation unchanged (Figure 8), the activation of CaMKII effectively rose during low frequency calcium stimulation. Moreover, the activated area of CaMKII was more than 14 times larger than that of calcineurin in the high frequency range (about 50 Hz to 200 Hz), which was more than 3 fold increase when compared with the ratio obtained in the low affinity simulation (Figure 8). This indicates that, although CaMKII activation outpaces PP1 activation at high frequency stimulation (Figure 4b), PP1 still plays an important role in shaping CaMKII response to calcium. The potency of PP1, which is controlled by DARPP-32, not only determines the sensitivity of the CaMKII frequency response, but also the extent to which CaMKII can be activated by high frequency calcium inputs. Because DARPP-32 is a hub of kinases and phosphatases [53], this points to avenues of controlling synaptic plasticity via different signaling pathways.

Combined with the result obtained above, the extent of which CaMKII can be phosphorylated on Thr286 determines the final activity of CaMKII during high-frequency calcium stimulation. Since autophosphorylation increases the affinity of calmodulin for CaMKII, these results can be further interpreted as the extent of CaMKII activity at high frequency calcium stimulation increases in proportion with the affinity between CaMKII and calmodulin.

### Calmodulin concentration is another regulator of the sensitivity to frequency

**Figure 9:**
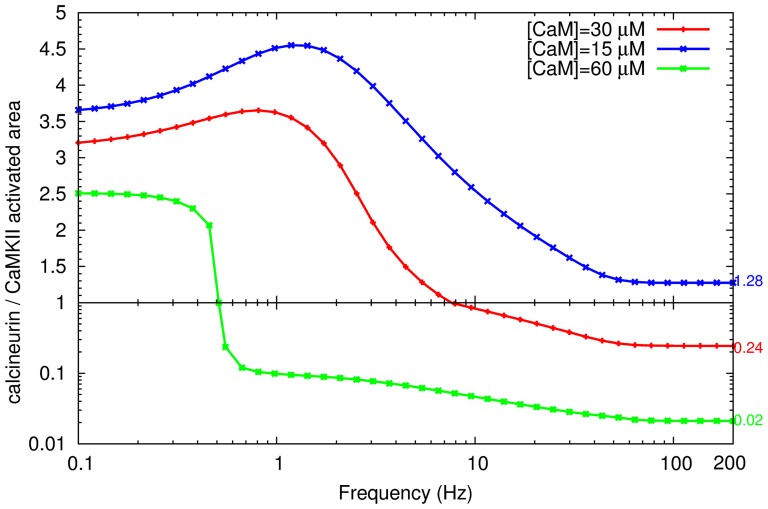
Effect of calmodulin concentration on the relative activation of calcineurin and CaMKII. Comparison of the relative activation between calcineurin and CaMKII, when different concentrations of calmodulin are available. Calmodulin concentration among different curves is different, but the number of calcium inputs and the amount of molecules for each input remain the same (100 inputs, 34560 molecules per input). On a given curve, each point shows the ratio of activated area of calcineurin versus CaMKII, when stimulated by a train of calcium inputs at a given frequency.

Calmodulin not only transfers its calcium-frequency sensitivity to CaMKII, but also regulates the final extent of CaMKII activity. As shown in Figure 9, when calmodulin concentration was increased from 30 micromolar to 60 micromolar, the amount of CaMKII activation increased, as the ratio between activated area of calcineurin and CaMKII decreased at all tested frequencies. On the contrary, when calmodulin concentration decreased to 15 micromolar, the activity of CaMKII decreased. Importantly, the extent of CaMKII activation at high calcium frequencies is positively regulated by calmodulin concentration. It therefore seems that calmodulin plays a dual role in the activation of CaMKII. Besides its ability to activate CaMKII subunits, the amount of calmodulin also limits the extent of CaMKII autophosphorylation on Thr286, in response to different frequencies. As calmodulin, that is available, is less abundant than CaMKII in the spine [60], the availability of calmodulin limits the chance of a CaMKII subunit having a neighboring subunit in the active state, able to phosphorylate it. If we view autophosphorylation and calmodulin trapping as a cooperative process, calmodulin availability would determine the input frequencies that induce the onset of this process.

## Discussion

The influx of calcium through NMDA receptors is of particular importance for synaptic plasticity. However, the mechanisms underlying the dual role of calcium, triggering either LTP or LTD, are still unclear. Several models have already investigated the effect of calcium on postsynaptic plasticity, but none of them accurately modeled calmodulin activation in detail, nor considered the existence of a phosphatase whose activity can be regulated by calcium as well. Furthermore, the correlation between the frequency of calcium inputs and the total amount of calcium ions has never been systematically studied before. In the present study, we explored how postsynaptic calcium elevation could drive different biochemical cascades by inducing different responses of calcineurin and CaMKII according to calcium input frequency, amplitude, and duration. We show that increased calcium input frequency increases both the activation of calcineurin and CaMKII. However, changing input frequency shifts the relative activation between the two enzymes. In addition, activation of CaMKII can be achieved either by high frequency calcium input, or at a low frequency, by stimulation with large input size.

In many ways, the results of our simulations are in agreement with previous experimental reports. Experimental evidence has shown that low frequency calcium inputs lasting longer induce strong activation of calcineurin. For instance, Mulkey *et al.* (1992) showed that the electrical induction of LTD resulting from low frequency afferent stimulation needs to last for 30 s [61]. Yang *et al.* (1999) showed that a moderate increase in calcium for about 1 min could induce LTD [14]. Short high frequency calcium inputs preferentially trigger the activation of CaMKII. This is also supported by experimental evidence showing that LTP is elicited by stimulation transiently raising calcium to high levels [62] for about 2 seconds [14], [63]. In our simulations, larger calcium input size allowed CaMKII activation at lower calcium input frequencies (Figure 7b). This has been demonstrated in experiments showing that low-frequency afferent stimulations paired with postsynaptic depolarizations result in LTP rather than LTD [64]. In addition, this computational model provided insights into the range of LTP-inducing frequencies of postsynaptic calcium spikes.

A major finding of the current research is the regulation of total amount of calcium ions on the frequency-sensitivity of synaptic plasticity. This may explain why the quantitative characteristics of postsynaptic calcium, required for triggering either LTP or LTD, have been difficult to determine. It is simply because there is no such absolute thresholding frequency or amount of post-synaptic calcium ions for synaptic changes in either direction. Rather, the synaptic connection is regulated based on a cell-type- or individual cell- specific manner. One important underlying evidence is that not all NMDA receptors are equivalent. The diverse compositions of NMDA receptors expressed in different cells, or in a single cell but at distinct developmental stages, confer various channel properties [65], [66]. This affects the transient property of calcium influx, therefore, controlling the induction of LTP or LTD. Furthermore, NMDA receptor subunit composition is also regulated by the excitation history of the spine [67]–[69], therefore previous activities can regulate further excitations.

High frequency stimulations induce short but potent elevations of free intracellular calcium concentration, and these specific calcium transients impose a specific temporal constraint for calcium decoding proteins, such as calmodulin. We show that calmodulin activation depends on calcium input frequencies. With a constant total input of calcium ions, high frequency signals stimulate calmodulin more efficiently.

Not only Calmodulin activates CaMKII, its availability also influences how CaMKII responds to calcium input frequencies (Figure 9) [70], [71]. This finding suggests the importance of calmodulin buffer proteins, such as neurogranin [72], on the regulation of synaptic plasticity, because calmodulin concentration is always a limiting factor [60].

Finally, there may be active recruitment processes for calmodulin taking place in specific locations within a spine, for instance, in the area near calcium channels in the post-synaptic-density. Calmodulin may be recruited from other calmodulin binding proteins to CaMKII, because of the increased affinity between CaMKII and calmodulin induced by high-frequency stimulation. Calmodulin may also be translocated via process mediated by actin filament, since synaptic activity can regulate actin polymerization [73]. According to our simulation results, calmodulin recruitment can actively reduce the calcium-spike frequency required for CaMKII activation. Furthermore, the more calmodulin is accessible, the longer CaMKII will be activated, which may play an important role in modulating synaptic plasticity.

It has often been believed that CaMKII-frequency sensitivity is due to its multimeric structure and intersubunit autophosphorylation. High frequency calcium pulses successfully induce the activation of large amounts of calmodulin, and this enhances its probability of binding to neighboring subunits of CaMKII, thus inducing CaMKII autophosphorylation. This autophosphorylation promotes high affinity calmodulin binding, thus CaMKII responds to higher frequency signals with a positive feedback. We demonstrate here that even without autophosphorylation on Thr286, CaMKII shows frequency sensitivity to a certain extent. This is mainly due to the activation of calmodulin. Because at higher calcium-input frequencies, more calmodulin is activated and more CaMKII subunit can therefore be activated for longer period. However, autophosphorylation deepens the extend of CaMKII activation at higher frequencies and increases the competition of CaMKII with calcineurin, therefore playing a determinant role on synaptic efficacy. It has been shown that the autophosphorylation of CaMKII on Thr286 can result in bistability of enzyme activity [74]. De Koninck *et al.* used *in vitro* experiments to demonstrate that CaMKII autophosphorylation occurs in a frequency-dependent manner and this frequency response is modulated by the amplitude and duration of each calcium pulse, which is congruent with our findings [37]. Although a higher calcium input frequency shifts the preferential activation from calcineurin to CaMKII, the total amount of calcium ions in the signal defines how sensitive the system is towards different calcium input frequencies. In other words, large amounts of calcium ions lower the frequencies that induce the autophosphorylation of CaMKII, as can be seen in Figure 7a and b. Thus, variation of calcium input frequency may be a strategy for neurons to build up transient calcium concentrations when total calcium ions in a stimulus are limited. The activation of CaMKII can be induced equally well either by a high frequency of calcium influx or a large amount of calcium ions. Interestingly, the range of frequencies sensitive to the activation of signal goes from 3 to 50 Hz, corresponding roughly to the frequencies used to induce postsynaptic LTD and LTP [61], [62].

The phosphorylation of threonine 305/306 (Thr305/306) of CaMKII was not considered in this model. This phosphorylation happens after the dissociation of calmodulin from CaMII while the kinase is still phosphorylated on Thr286, and it prevents calmodulin binding [75], [76], thus acting as a desensitization mechanism [77]. Since the phosphorylation on Thr286 increases the affinity between calmodulin and CaMKII, the dissociation of calmodulin and subsequent phosphorylation on Thr305/306 are very slow processes [75]. The phosphorylation on Thr305/306 has been shown to be strongly related to induction of LTD and this induction is calcineurin dependent [78]. Based on the fact that this phosphorylation site will not affect the kinase activity within the time range we considered, we decided to not address this phosphorylation site in this model.

LTP and LTD are two large classes of phenomena regulating the weight of possibly all excitatory synapses. However, their underlying mechanisms vary depending on the synapses and the neuronal circuits involved [79]. This model can be further applied to different type of neurons in different circuits. For instance, in the medium spiny neuron of the striatum (MSN), dopamine afferents modulate glutamate induced synaptic plasticity through DARPP-32 inhibition of PP1, thus regulating CaMKII. This control on neuronal plasticity is at the heart of reward mechanisms and drug addiction [80]. DARPP-32 is highly expressed in MSNs, and its high reported affinity for PP1 means that inhibition is almost irreversible. As shown above (Figure 8), this inhibition increases CaMKII sensitivity towards low frequency calcium firing. Accordingly, the activation of dopamine D1 receptors and the DARPP-32 pathway can trigger LTP in MSNs, as shown by Wolf *et al.* [81]. Furthermore, repeated *in vivo* treatments with psychostimulants increases the surface expression of AMPA receptors in the striatum [82]. However, paradoxically, a single cocaine injection in drug-naive animals exerts no effect on synaptic plasticity, while in drug-experienced animals it induces LTD [83]. This may suggest a more complicated process underlying long term neuroadaptation and drugs of abuse.

This model can also be adapted to understand the effect on synaptic efficacy of calcium influx through other receptors. If a sharp, high amplitude increase of free calcium reduces the requirements on high-frequency stimulation for activating CaMKII, while a moderate calcium influx requires much higher frequencies to build up, then a delayed but prolonged calcium increase induced by metabotropic glutamate receptor (mGluR), especially mGluR1 and mGluR5, through activation of intracellular calcium stores, is more likely to induce LTD [79], [84]. A future challenge for this model will be to understand, when several mechanisms for increasing intracellular calcium concentration are simultaneously activated, the response of downstream signaling pathways.

The computational model presented here improves our understanding of calcium signaling involved in synaptic plasticity. The frequency of postsynaptic calcium influx regulates the induction of LTP and LTD, while the amount of calcium ions shifts the windows of frequencies required for this bidirectional regulation. Besides, the availability of calmodulin and the phosphorylation on Thr286 of CaMKII not only regulate the frequency sensitivity but also the extent of CaMKII activity at high calcium frequencies. Furthermore, synaptic plasticity is induced in a cell-specific manner, and is modulated by other pathways, such as the dopamine regulated PP1 inhibition in MSN.

## Methods

### Model Structure and Validation

The model encoded in the XML format used by E-Cell3 is provided as Description S1.

**Figure 10:**
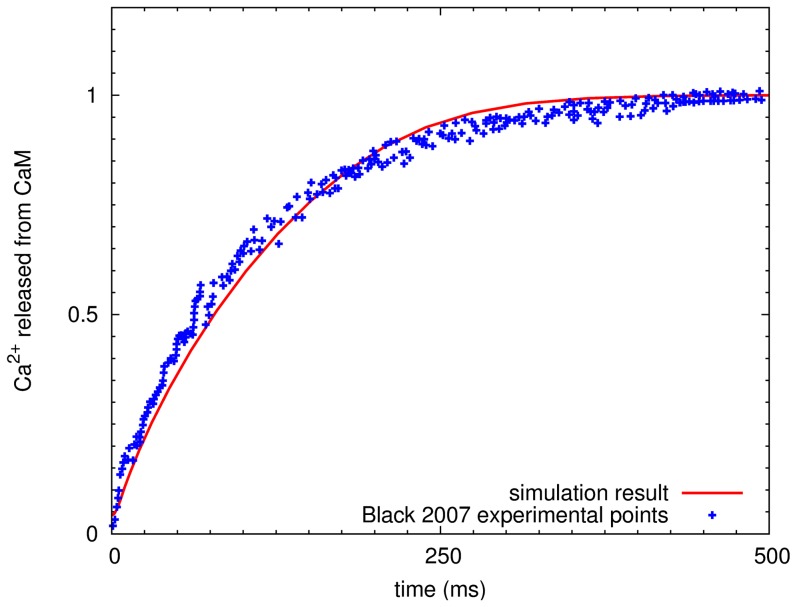
Validation of calcium and calmodulin binding kinetics. Calcium ions released from calmodulin, normalized by the maximum capacity of release. Crosses, experimental data from Black [85]. Solid line, simulation result. The dissociation of calcium from calmodulin was initiated by adding fluorescent calcium chelator quin-2 in the model as described in the corresponding experiment (initial conditions: 4 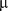
M calmodulin, 50 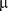
M calcium and 400 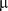
M quin-2). Only the first two released calcium ions were measured as the dissociation of the two additional calcium ions were not measured in this experiment.

The activation of calmodulin by calcium was modeled as described previously [20]. In this model, calmodulin exists under two states in thermal equilibrium, the open (R) and the close (T) state. In either state, calmodulin can bind up to four calcium ions. Each calcium binding site is considered unique, with its own specific dissociation constants, different in the R and T states. Calmodulin can undergo transitions between R and T state, regardless of the number of calcium ions bound. Because its affinity for the R state is higher than for the T state [20] (where the connection between affinity and free energy in the calmodulin example is discussed), binding of calcium progressively lowers the free energy of the R state, facilitating the transition from T to R state. Once calmodulin is in the R conformation, it can bind to target proteins, calcineurin and CaMKII in the model, and activate them. The transient dynamics of calcium association and dissociation with calmodulin was justified by stopped-flow fluorescence measurements [85]. This validation procedure was achieved before adding calcium pumps, buffer proteins, and other signaling molecules (Figure 10).

**Figure 11:**
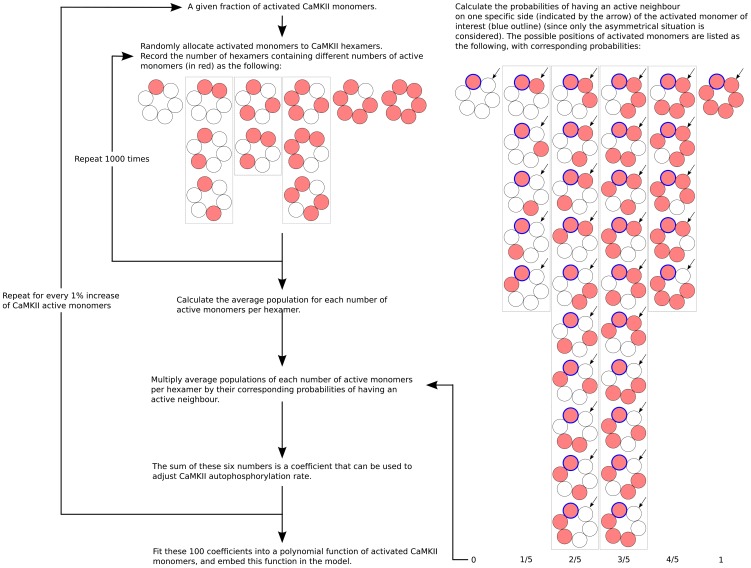
Calculation of the rate of CaMKII autophosphorylation. The procedure for calculating the rate of CaMKII autophosphorylation as a function of active CaMKII monomers (for details see Methods section).

**Figure 12:**
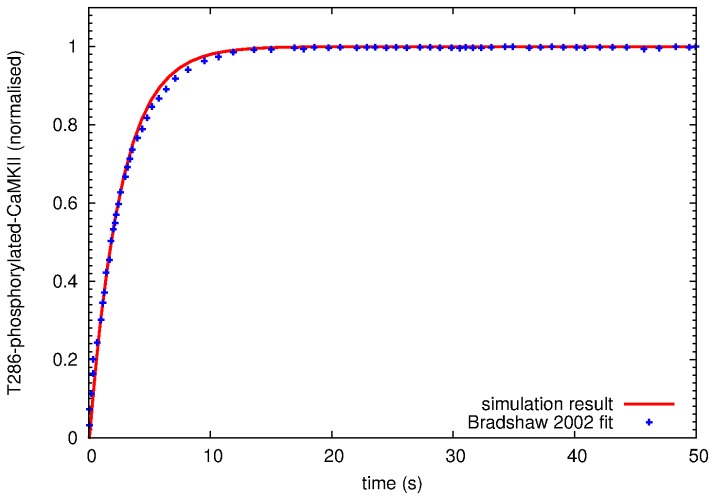
Validation of autophosphorylation on Thr286 of CaMKII. Time course of CaMKII autophosphorylation on Thr286. Points, experimental data measured in the presence of abundant ATP from Bradshaw et al. [86]. Solid line, simulation result with initial concentrations as described in the experiment (500 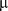
M calcium, 10 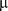
M calmodulin, and 0.4 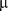
M CaMKII). CaMKII autophosphorylation is initiated by quickly mixing the enzyme with calcium and calmodulin both in the experiment and the model.

We extended the model described above with a detailed description of the processes controlling CaMKII autophosphorylation. In our model, only monomeric subunits of CaMKII were considered as physical species, and autophosphorylation was modelled as a first order reaction following calmodulin binding. In order to accurately model autophosphorylation on Thr286 within the context of the hexamer and take into account the fact that this is a trans-phosphorylation between adjacent subunits in a holoenzyme [33], we computed a correction of the rate based on the probability that monomers have active neighbors. We assumed the autophosphorylation of CaMKII occurs within the hexameric ring of the holoenzyme, and happens in an asymmetric manner. We proceeded in two steps. First, for each number of active subunits per hexamer, we computed the probability for a given subunit of having an active neighbor (Figure 11, right). Then, we set up a random simulator to calculate the distributions of active CaMKII subunits within hexamers as a function of the total amount of active subunits (Figure 11, left). In order to achieve this, for a given quantity of active CaMKII monomers, we randomly assembled them with inactive monomers into hexamers, and recorded the number of hexamers containing one active subunit, two, three, and so on. After 1000 repeats of this random simulation, the average fraction of each number of active monomers per hexamer was multiplied by the corresponding probability of having an active neighbor, as computed in the first step. The sum of these products of multiplications was then used as a coefficient to adjust the autophosphorylation rate. The whole procedure was repeated for every 1% increase of active monomers. Finally, these 100 generated values were fitted by a polynomial function of degree 5. This polynomial function was embedded in the model, dynamically changing the autophosphorylation rate at each simulation step (the detailed procedure for this calculation is illustrated in Figure 11, the fitted polynomial function is plotted in Figure S3). Once CaMKII was phosphorylated, its affinity for calmodulin increased in the model, as reported by Meyer *et al.* [36]. The inter-holoenzyme autophosphorylation of CaMKII on Thr286 was in good agreement with the experimental time course obtained from chemical quenched flow kinetics under corresponding experimental conditions [86] (Figure 12).

**Figure 13:**
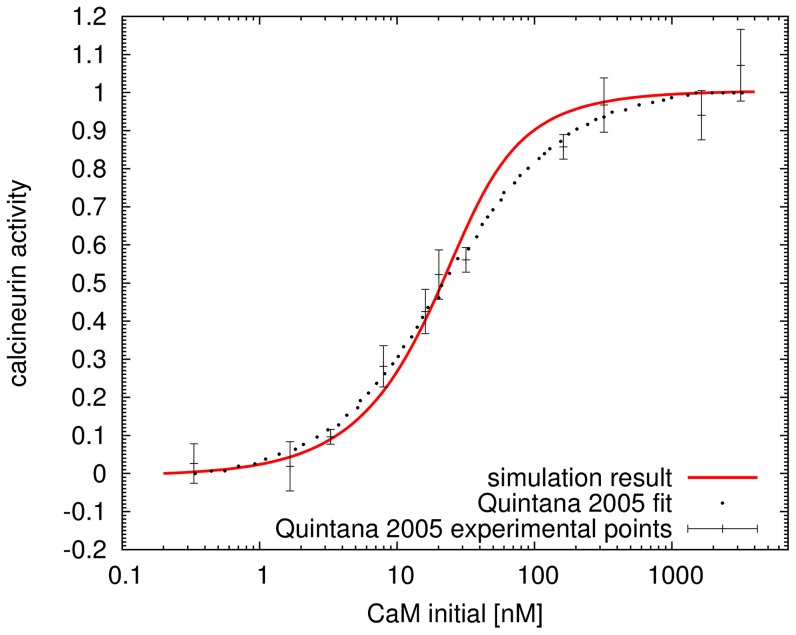
Validation of binding and dissociation rates of calcium and calmodulin to calcineurin. The normalized activation of calcineurin. Points with error bars, experimental observation from Quintana [87]. Dashed line, curve fitted by Quintana. Solid line, simulation result. Steady state results of stimulation at different initial concentrations of calmodulin. The concentration of calcineurin is 25 nM, and that of calcium 100 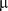
M.

We modeled the activation of calcineurin by sequentially binding four calcium ions first, which activates its regulatory subunit and permits the association of its catalytic subunit with Ca^2+^/calmodulin [21]. The binding rates of calcium ions and calmodulin to calcineurin were verified by an assay measuring calcineurin activation by calmodulin in the presence of calcium [87](Figure 13). Calcineurin binds calmodulin with higher affinity than CaMKII [87], [88], which facilitates its sensitive response to calcium mediated synaptic stimulation [89]. Active calcineurin dephosphorylates DARPP-32 on threonine 34 (Thr34) [59].

In our model, approximately 40% of DARPP-32 was activated by the basal level of cyclic AMP-dependent protein kinase (PKA). These phosphorylated DARPP-32 molecules, in turn, inhibited almost 90% of total PP1. The inhibition was modeled following a competitive inhibition mechanism. Only phosphorylation on Thr34 of DARPP-32 was considered in this model.

The calcium efflux was modeled as a pump, using Michaelis-Menten kinetics. The calcium influx was modeled by a zero-order reaction with a fixed rate. The large number of calcium buffer proteins present inside the spine were modeled as four binding reactions with different speeds, but the same affinity [49].

### Parameter definition: the activated area

**Figure 14:**
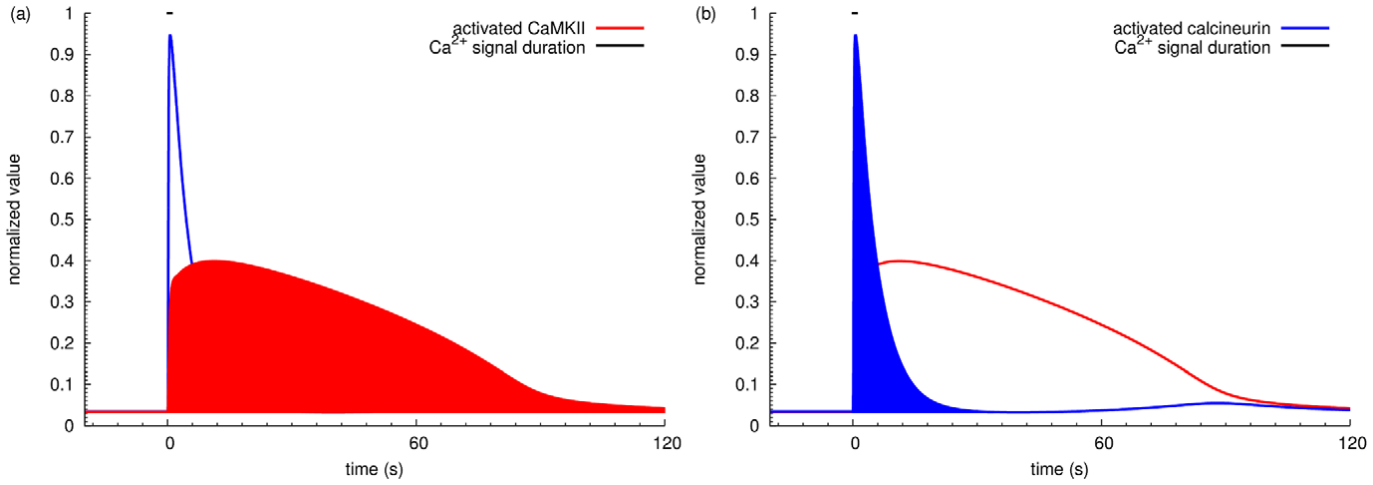
Definition of “activated area”. The activated areas for CaMKII in blue and calcineurin in red are represented in a) and b) respectively. The time courses represent active calcineurin and CaMKII upon stimulation by a train of calcium inputs at 50 Hz.

The activation of calcineurin and CaMKII by calmodulin displays distinct temporal characteristics. For the purpose of representing effective enzyme activity [53], we integrated the concentration of activated calcineurin and CaMKII over time, and calculated the area above the basal level of activity. This is defined as the “activated area” as shown in Figure 14. Specifically, the area within every simulation step was approximated as a trapezium. Then, the activated area was computed as the sum of all the trapeziums along simulation time minus the area of basal activity (basal level activity multiplied by simulation time). This area represents both the duration and the amplitude of enzyme stimulation and, since catalytic activity is considered constant, this effectively reflects the amount of substrate which will be affected. The ratio between the activated area of calcineurin and CaMKII was computed as a parameter to judge the preferential activation on calcineurin and CaMKII after calcium stimulation at a specific frequency.

### Modeling and simulation software

The model was based on the description of biochemical processes using continuous variables, that were simulated with a deterministic method. Modeling and simulation were performed using the E-Cell system, version 3 [90]. E-Cell is a module based, object-oriented simulation environment suitable for the modeling, simulation and analysis of large scale cell biological models. E-Cell defines the simulation model as a set of objects connected to each other. A biochemical reaction can be built by connecting variable, process, and stepper objects, where a variable represents a molecular species (entity pool), a parameter or a container, a process represents the kinetic law that results in changes in the values of one or several variables, and a stepper attaches a specific simulation algorithm to a set of processes. E-Cell supports various stepper functions, and can incorporate different algorithms and time scales into one model via its unique discrete integration meta-algorithm.

A generic ODEStepper, based on combined Radau 5 and Dormand-Prince 5(4)7M [91] algorithms, was used for the elementary reactions. E-Cell switches between these two algorithms depending on the stiffness of the system of equations at a given time point. Simulations were performed on the computing cluster of the European Bioinformatics Institute, which is composed of Intel-based nodes under GNU/Linux. The simulations were launched concurrently, with each simulation for a specific calcium firing frequency running on an individual core.

### Reactions and parameters

Models are based on a single homogeneous compartment of 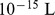 in volume, representing the spine [92]. The full model comprises 648 reactions. Reactions were modeled with Mass Action Law processes. Each enzymatic reaction was represented by the three elementary steps of binding, dissociation, and catalysis. The association (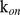), dissociation (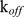), and catalytic (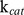) constants were mainly obtained from published information and are listed in Table 1.

**Table 1:** List of parameters used for simulation of the model.

Parameter	value	reference
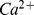 binds 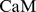 :		
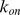	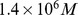 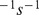	[20]
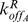	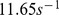	[20]
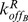	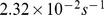	[20]
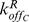	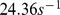	[20]
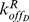	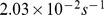	[20]
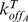	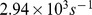	[20]
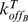	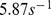	[20]
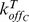	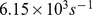	[20]
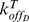	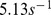	[20]
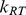	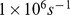	[20]
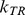	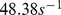	[20]
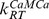	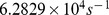	[20]
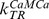	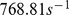	[20]
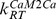	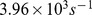	[20]
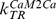	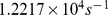	[20]
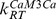	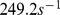	[20]
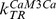	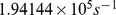	[20]
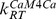	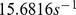	[20]
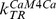	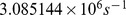	[20]
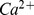 binding calcium buffer protein ( 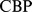 ):		
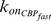	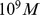 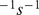	[93]
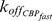	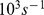	[93]
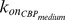	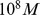 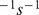	[93]
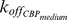	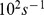	[93]
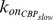	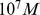 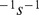	[93]
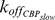	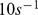	[93]
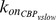	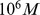 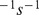	[93]
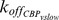	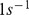	[93]
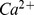 pump:		
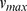	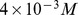 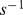	[93]
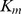	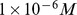	[93]
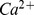 leak:		
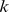	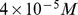 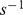	[93]
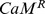 binding substrates:		
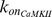	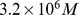 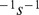	[88]
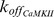	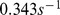	[88]
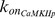	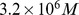 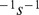	[88]
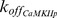	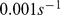	[36]
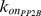	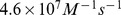	[87]
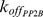	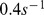	[94]
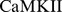 autophosphorylation on 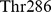 :		
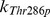	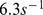	[95].This parameter is further refined during each simulation run.
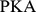 phosphorylates 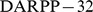 on 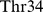 :		
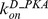	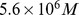 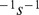	[96]
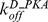	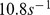	[96]
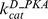	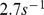	[96]
Calcineurin ( 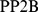 ) dephosphorylates 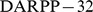 on 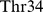 :		
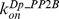	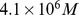 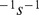	[97]
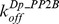	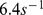	[97]
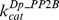	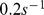	[97]
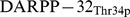 binding 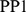 :		
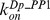	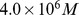 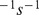	[56]
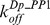	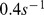	[56]
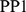 dephosphorylates 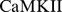 :		
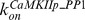	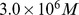 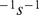	this study
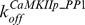	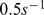	this study
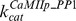	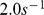	[74]
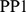 dephosphorylates 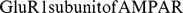 :		
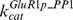	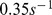	[98]
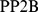 dephosphorylates 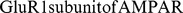 :		
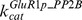	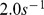	[98]
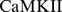 phosphorylates 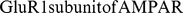 :		
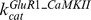	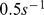	[98]
Concentrations:		
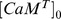	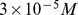	[99]
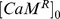	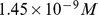	[20]
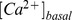	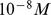	estimated from [100]
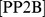	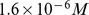	[101]
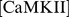	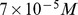	[102]
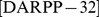	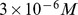	[26]
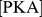	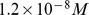	estimated from [103]
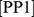	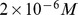	[104]
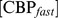	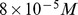	[49]
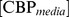	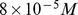	[49]
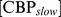	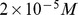	[49]
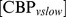	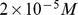	[49]
Compartment:		
spine volume	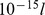	[92]

### Pathway activation

Calcium inputs were implemented using Python scripts, consisting of repeated increases of the calcium influx constant of a zero-order reaction. A typical simulation file can be found in Description S2. The duration for each increase was 8 milliseconds. Throughout most of the study, the number of calcium ions inserted in the system at each input was 34560 molecules. To study the effect of input size, the number of calcium ions for each input was increased accordingly. The delay before the next calcium input varied according to the frequency. However, when the input frequency was too high to have the time interval between two spikes longer than 8 milliseconds, the influx constant and opening duration would be recalculated in order to keep the number of calcium ions in each input constant. For instance, a 50 Hz signal was composed of a train of calcium inputs, each of which lasted 8 milliseconds, and a 12 milliseconds interval between each pair of inputs. However, at 200 Hz, the delay between each opening was 5 milliseconds. Since this was below the 8 milliseconds threshold, the system considered the opening was 5 milliseconds, and calculated the new influx constant based on this new opening time.

## Supporting Information

Description S1 **Model description.** All model reactions encoded in the XML version of the E-Cell3 modeling language.
(EML)

Description S2 **Simulation description.** Description of the simulation procedure encoded in the Python programming language.
(PY) Click here for additional data file.

Figure S1 **Intracellular free calcium concentration increase induced by stimulation inputs.** Increase of postsynaptic free calcium concentration triggered by a train of calcium inputs. Two specific input frequencies are shown here: 6 inputs at 0.98 Hz (red line), 100 inputs at 52.8 Hz (blue line).
(TIFF)

Figure S2 **Intracellular free calcium concentration increase induced by a single stimulation input.** Increase of postsynaptic free calcium concentration induced by various inputs at different amplitudes. Following a single calcium input with different input sizes, the peak amplitudes, are achieved within 10 milliseconds, followed by rapid decay back to basal level.
(TIFF)

Figure S3 **The fitted polynomial function.** The polynomial function fitted by 100 corrections for the rate of CaMKII autophosphorylation. The 100 corrections are calculated in terms of the distribution of activated CaMKII monomer and the probability of having an active neighbor (details see methods section).
(TIFF)
