# Supplementary figures and images for: Calcium Input Frequency, Duration and Amplitude Differentially Modulate the Relative Activation of Calcineurin and CaMKII
Source: PLoS One. 2012 Sep 4;7(9):e43810. doi: 10.1371/journal.pone.0043810 (PMC3433481; doi:10.1371/journal.pone.0043810)

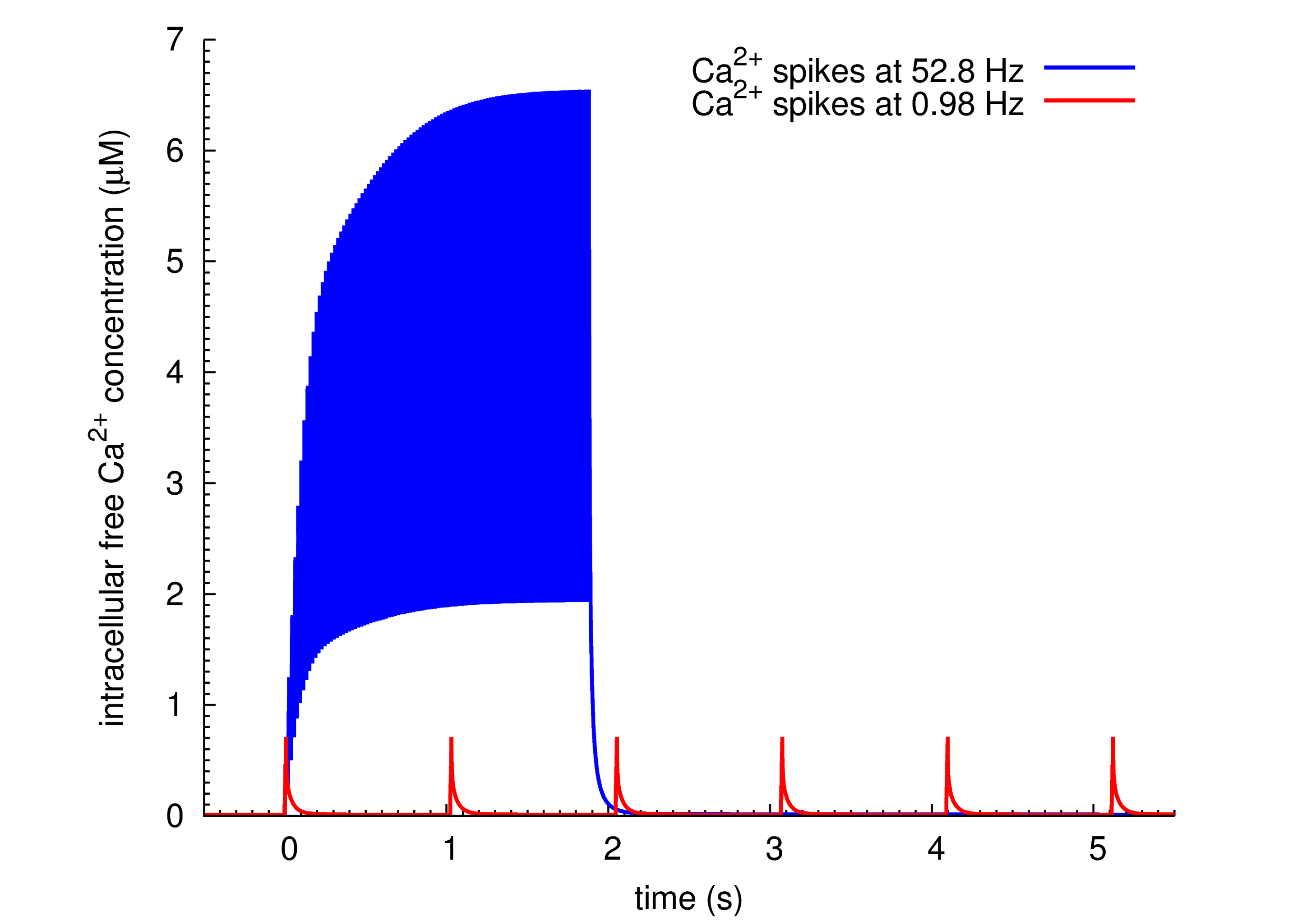

Supplement: Figure S1 — Intracellular free calcium concentration increase induced by stimulation inputs. Increase of postsynaptic free calcium concentration triggered by a train of calcium inputs. Two specific input frequencies are shown here: 6 inputs at 0.98 Hz (red line), 100 inputs at 52.8 Hz (blue line). (TIFF) [file pone.0043810.s003.tiff]

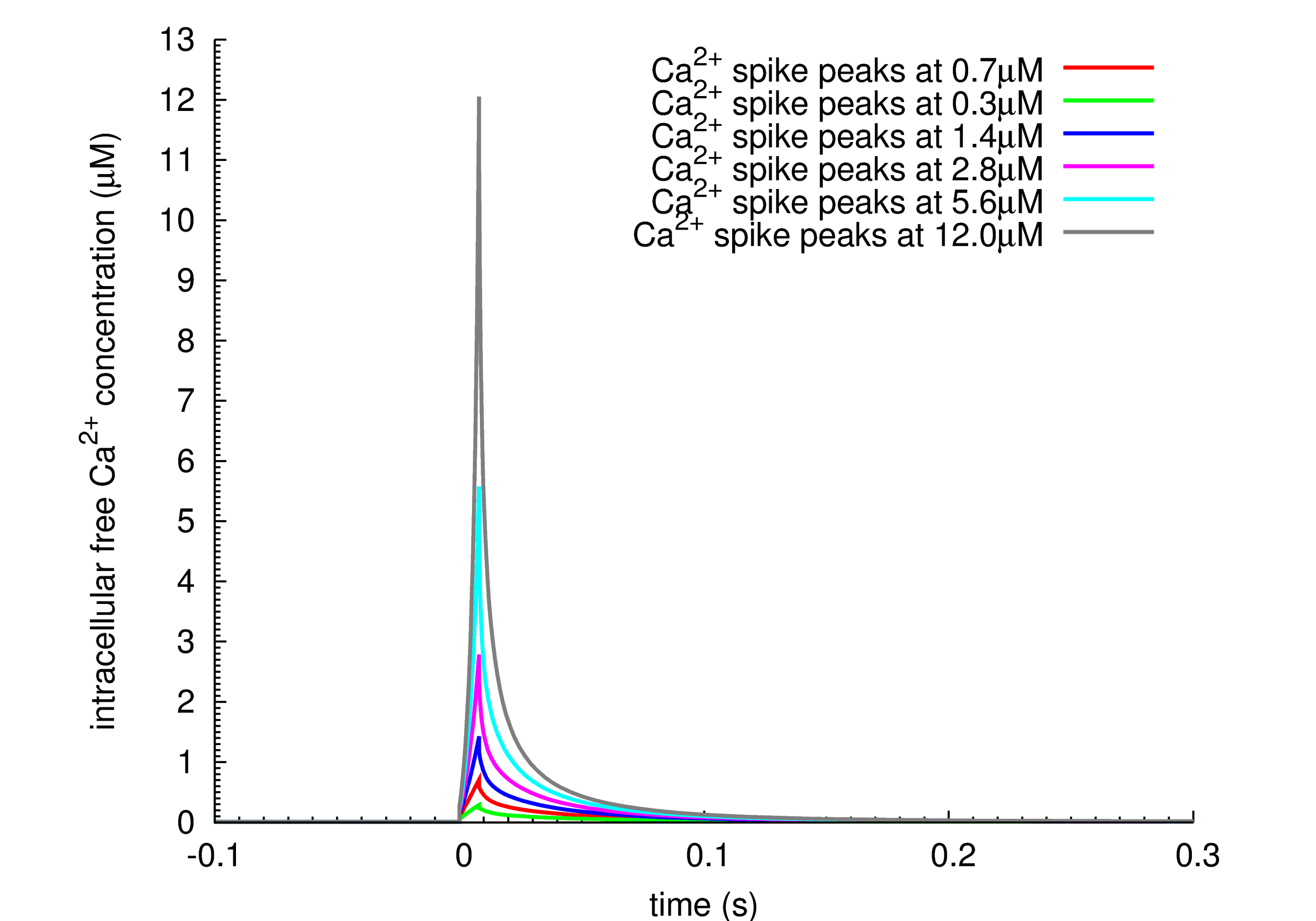

Supplement: Figure S2 — Intracellular free calcium concentration increase induced by a single stimulation input. Increase of postsynaptic free calcium concentration induced by various inputs at different amplitudes. Following a single calcium input with different input sizes, the peak amplitudes, are achieved within 10 milliseconds, followed by rapid decay back to basal level. (TIFF) [file pone.0043810.s004.tiff]

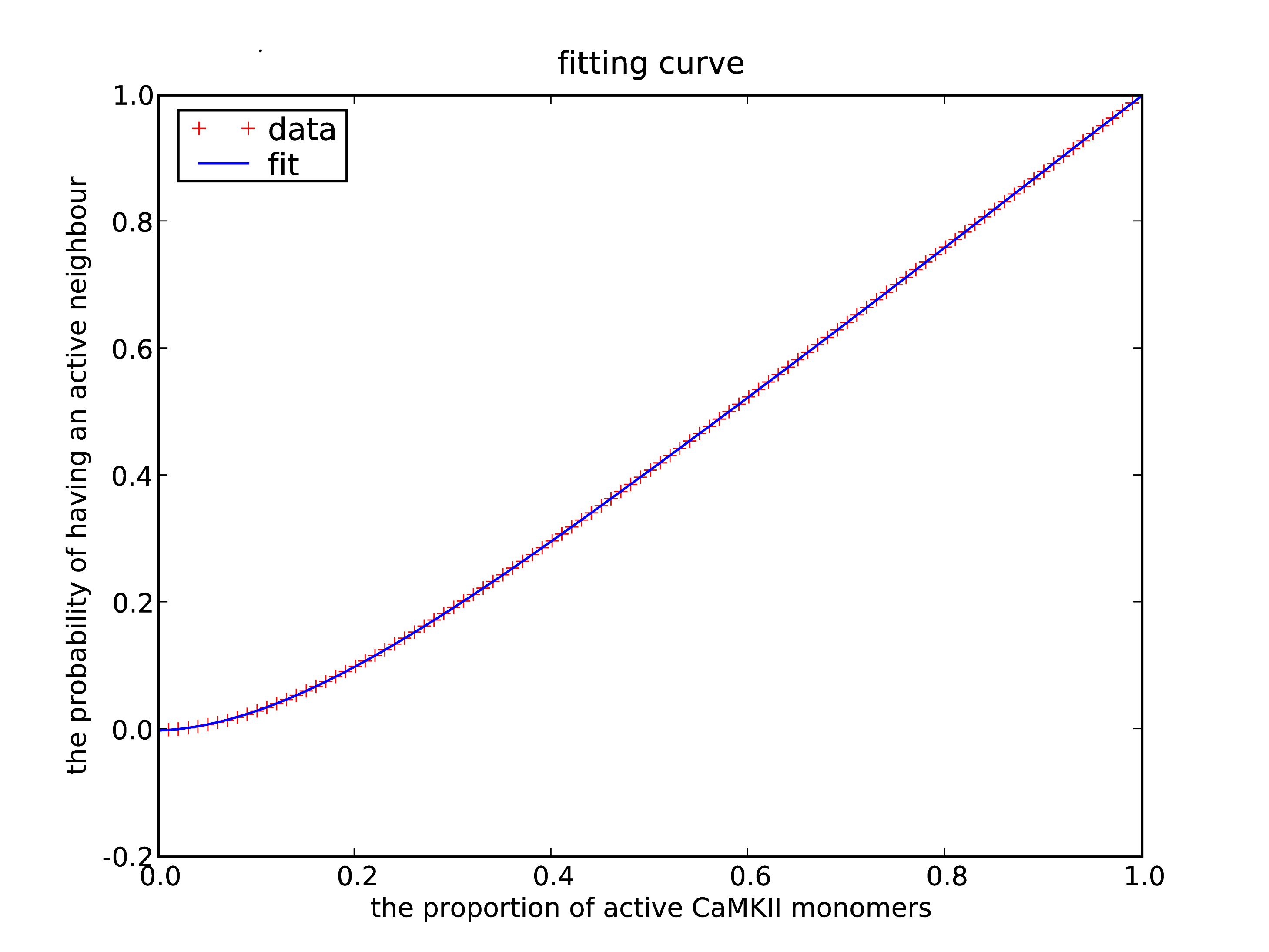

Supplement: Figure S3 — The fitted polynomial function. The polynomial function fitted by 100 corrections for the rate of CaMKII autophosphorylation. The 100 corrections are calculated in terms of the distribution of activated CaMKII monomer and the probability of having an active neighbor (details see methods section). (TIFF) [file pone.0043810.s005.tiff]
